# Prevention of *Cryptosporidium* and *GIARDIA* at the Human/Gorilla/Livestock Interface

**DOI:** 10.3389/fpubh.2018.00364

**Published:** 2018-12-14

**Authors:** Gladys Kalema-Zikusoka, Stephen Rubanga, Birungi Mutahunga, Ryan Sadler

**Affiliations:** ^1^Conservation Through Public Health, Entebbe, Uganda; ^2^Bwindi Community Hospital, Kanungu, Uganda; ^3^School of Veterinary Medicine, University of California, Davis, Davis, CA, United States

**Keywords:** Bwindi, *Cryptosporidium*, disease transmission, *Giardia*, mountain gorilla, One Health, Uganda

## Abstract

Mountain gorillas (*Gorilla beringei beringei*) are critically endangered and found in Bwindi Impenetrable Forest and Virunga Volcanoes. Habitat destruction, high human population growth rates, poverty, and disease are threatening the survival of mountain gorillas. A study implemented in 2010 investigated the prevalence of *Cryptosporidium* and *Giardia* sps., as part of a long-term gorilla health-monitoring program at Bwindi through regular fecal sample collection, and comparative pathogen analysis at the human/gorilla/livestock interface. Samples collected from habituated and non-habituated gorillas, community-owned livestock herds and people at Bwindi were screened for *Cryptosporidium* and *Giardia* sps. using ImmunoSTAT Commercial Field Kit and doubtful samples confirmed with Direct Fluorescence Antibody Test (DFA). *Giardia* was found in 5.5% of livestock, 40% of symptomatic humans from the local hospital and 9.5% of asymptomatic park staff, but not in gorillas. *Cryptosporidium* was found in 3.1% of habituated gorillas, 4.7% of livestock, and 62.4% of park staff. Whereas, previous studies have compared *Cryptosporidium* and *Giardia* sps. in gorillas and livestock, this is the first study making a comparison in humans, gorillas and livestock. Unlike previous studies in Bwindi and Virungas, no *Giardia* sp. was found in gorillas. The improving hygiene and sanitation of local communities sharing a habitat with gorillas through Village Health and Conservation Teams (VHCTs) established in 2007, could have contributed to the decreased prevalence of *Giardia* in this mountain gorilla population. *Cryptosporidium sp*. only found in the habituated gorillas could be associated with human interaction, similar to previous studies. A subsequent VHCT was selected for each village with positive human samples and where gorillas often range, local health centers were mobilized to educate patients on the health risks of collecting water from unprotected sources and cattle water troughs were built. This paper describes a One Health approach to reducing cross species disease transmission at the human/gorilla/livestock interface.

## Introduction

The IUCN status of Mountain gorillas has changed from critically endangered to endangered with a recent population estimate of 1,004 (1), found in two distinct populations: Bwindi Impenetrable Forest in Uganda, and the Virunga Volcanoes in Rwanda, Democratic Republic of Congo (DRC) and Uganda. Human poverty and high human population growth rates affect environmental sustainability, including survival of gorillas, through habitat destruction due to a greater need for firewood and food from the gorillas' habitat (2, 3) and through increased infectious disease incidences in poorer households that come into direct contact with gorillas ranging in their gardens. At Bwindi Impenetrable National Park and in other remote locations in Uganda bordering protected areas, family sizes often range from 8 to 10, and as such are less able to provide basic modern healthcare to all their children. Furthermore, due to lack of education for themselves and their children, they are less able to implement appropriate hygiene measures such as having hand washing facilities outside the toilet or pit latrine. Consequently when gorillas range in their homes, they are more likely to contract preventable infectious diseases from humans. Promoting family planning enables them to start breaking the poverty cycle leading to better health outcomes for themselves, their local community and potentially the mountain gorillas who they share the fragile habitat with (4).

These biodiversity hotspots provide critical habitat for gorillas and other endangered species including chimpanzees and elephants, and are surrounded by human population densities ranging from 300 to 600 people per square kilometer. Bwindi Impenetrable National Park was gazetted in 1991 and gorilla tourism began in 1993, bringing an end to commercial logging, but leaving a hard edge with most of the park having no buffer zone with the surrounding local communities. Gorillas frequently leave the park to forage on banana plants and eucalyptus trees found on community land and sometimes on other plants in community household gardens (4). In 1996 and 2000/2001, scabies disease outbreaks occurred in the gorillas. In 1996, the outbreaks occurred in Buhoma, the northern sector of the park, resulting in the death of an infant and morbidity in the other three individuals in the group that only recovered with Ivermectin treatment (5). In 2000, another scabies outbreak occurred in a second gorilla group in the southern sector of the park, affecting 17 gorillas, and though there was severe alopecia in some infant gorillas, they were all able to recover with Ivermectin treatment. The source of the scabies was eventually traced to local human communities with limited access to basic health and other social services (6).

There have been studies of intestinal helminth parasites in Bwindi gorillas (7, 8). Gastrointestinal disease is a cause of morbidity in gorillas, but not a primary cause of gorilla mortality (9), and in many cases may be asymptomatic. However, gastrointestinal parasites may have long-term effects for general health, survival, and reproduction of gorillas and other species (10). Furthermore, continued interaction and contact with humans and livestock may result in altered transmission rates and virulence of gastrointestinal helminths parasites for primates (11) and may well provide a route of transmission for other pathogens including *Cryptosporidium* and *Giardia* (12)

Conservation Through Public Health (CTPH), a non-governmental organization and non-profit, was founded in 2003, to address scabies and other zoonotic diseases at the human/gorilla/livestock interface. CTPH promotes biodiversity conservation by enabling people, wildlife and livestock to coexist through improving their health and livelihoods in and around protected areas in Africa and has three integrated programs: wildlife health and conservation, community health and alternative livelihoods. As part of setting up a long term gorilla health monitoring program to provide information to Uganda Wildlife Authority for timely wildlife management, CTPH built a Gorilla Research Clinic at Buhoma, Bwindi's main tourist site, which analyzes abnormal and monthly fecal samples from habituated gorilla groups to test for intestinal helminths parasites, as well as, comparative pathogen analysis with people and livestock through specific studies (13).

*Cryptosporidium parvum* and *Giardia duodenalis* are not naturally occurring in rivers, but can survive in the cyst stages in water and the environment following fecal contamination from humans and other mammals (14). They both infect a wide range of hosts, and can cause disease in their hosts as well as zoonotic disease (15). Symptoms of *Cryptosporidium sp*. infection include diarrhea, abdominal pain, nausea, vomiting and low-grade fever, and immunodeficient and malnourished patients are considered more susceptible to cryptosporidiosis. Symptoms of giardiasis include gastrointestinal disturbance, flatulence, diarrhea, and discomfort. Giardiasis tends to have more severe clinical signs than cryptosporidiosis, which is more of a problem in infants and immunocompromised individuals. Emerging resistance, climate change, and population growth are predicted to increase both malnutrition and the prevalence of these parasites in water sources (16).

Both *Cryptosporidium* and *Giardia* sps. have been recorded in mountain gorillas, people and livestock at Bwindi (17–20) and Virunga Massif (21, 22) and other wildlife species including buffalo that share a habitat with mountain gorillas in Virunga (22). Species of Cryptosporidium affecting livestock include *Cryptosporidium parvum* a common cause of neonatal diarrhea in bovine and caprine species, and *C. bovis, C. ryanae*, and *C. andersoni*, which tend to be found in larger calves and are non-symptomatic. *Cryptosporidium parvum, C. muris* and *C. meleagridis* (23, 24) have been found In mountain gorillas and *C. bovis* has been found in captive western lowland gorillas (12).

Though several species of Cryptosporidium and Giardia have caused clinical signs in humans and livestock (16), clinical signs in mountain gorillas because of either pathogens have not yet been documented.

Various methods have been used to test for these pathogens including conventional staining, fluorescein isothiocyanate–conjugated monoclonal antibodies (22); and immunofluorescent antibody (IFA) (17). However, the effectiveness of the simpler ImmunoSTAT, Commercial Field Kit has not been evaluated in mountain gorillas and livestock. This study assessed the feasibility of this fecal antigen Enzyme-Linked Immunosorbent Assay (ELISA) kit for interface disease investigation, as it is commonly used in the human health sector with curative, preventive, rehabilitative, and palliative care.

Though *Cryptosporidium* and *Giardia* have been documented in mountain gorillas, livestock and humans, no other study has looked at their regional occurrence in humans, gorillas and livestock to indicate possible fecal cross-contamination and institute measures to prevent further transmission.

This study also assessed a practical test used routinely in human medicine to detect and further prevent zoonotic disease transmission. We tested the feasibility of using simpler ImmunoSTAT tests as a routine screening tool in human medicine to detect zoonotic disease transmission of *Cryptosporidium parvum* and *Giardia intestinalis* between people, gorillas and livestock in low resource settings and using the manufacturers protocol to test doubtful ImmunoSTAT results using Direct Florescent Antibody (DFA) tests.

Results from the study were used to recommend and implement management actions in the wildlife, human and livestock health sectors to minimize disease transmission at the human/gorilla/livestock interface.

## Methods

All human specimens were collected and used in this study under guidelines established in Institutional Review Board approved protocols of the Bwindi Community Hospital. The animal research was approved by the University of California Davis STAR program committee, and Morris Animal Foundation who awarded a grant and complied with the legal requirements of Uganda and adhered to the research protocol submitted to conservation authorities. Permission to collect the fecal samples from gorillas was obtained from Uganda Wildlife Authority. Since the collection of fecal samples from gorillas was non-invasive and did not cause any observable distress to the animals, no animal ethic committee was consulted regarding our study. The gorilla fecal sampling was performed during routine health checks by Conservation Through Public Health employees through an MOU with UWA. Sampling from livestock was with full consent from farmers as part of livestock health checks through MOUs between CTPH and Kanungu District Local Government in Uganda.

**Figure 1:**
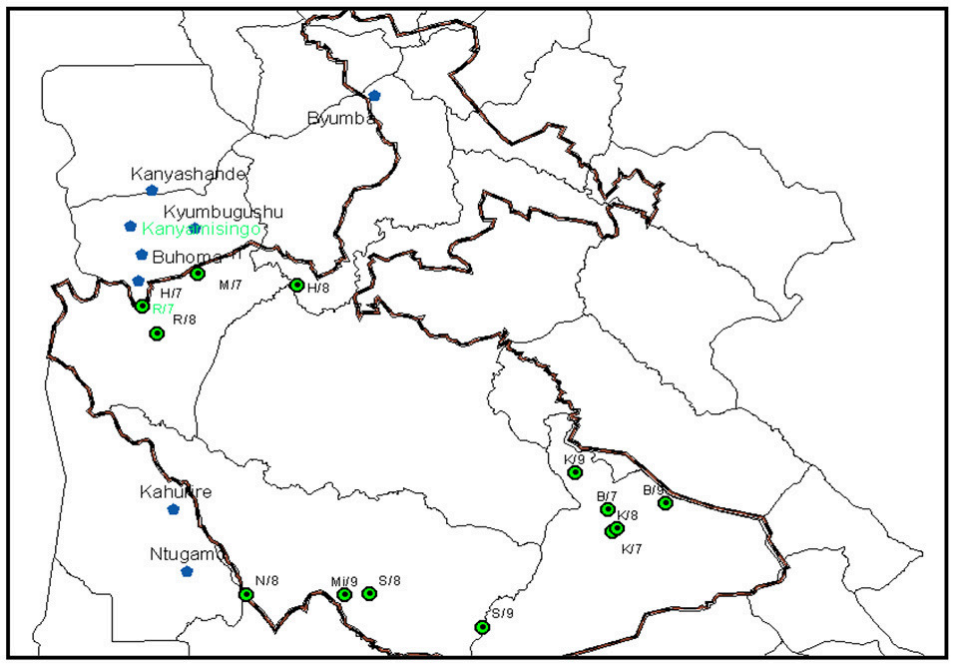
Linking the gorilla (green) and livestock (blue) data points where samples were collected using GPS.

Multistage cluster sampling by sector and family group was conducted to obtain a similar number of samples from each sector while collecting a proportionate number of samples per family group for (i) gorillas, both habituated gorillas during routine fecal sample collection for gorilla health monitoring and unhabituated gorillas during the 2011 gorilla census; (ii) livestock in Bujengwe and Mukono parishes, which have high human and gorilla conflict; (iii) and humans, where targeted sampling comprised of people presenting for clinical diarrhea from Bwindi Community Hospital (BCH), and staff working with gorillas from the Institute of Tropical Forest Conservation (ITFC) and Bwindi Impenetrable National Park (BINP) as part of a pilot Employee Health Program conducted by CTPH. Figure 1 shows GPS locations of gorilla and livestock sample collection sites.

Samples were collected from night nests of 97 individual habituated gorillas and 50 individual unhabituated gorillas; from the rectum of 127 individual livestock from Mukono and Bujengwe parishes at Bwindi, and fresh fecal samples from 106 individual humans including five clinical cases, who were all infants from BCH, 27 asymptomatic research staff from ITFC and 74 asymptomatic tourism and law enforcement staff from BINP.

### Gorilla Fecal Sample Collection

Samples were collected in fecal collection pots from fresh nests of the previous evening (<24 h from defecation). Each gorilla sample was identified if possible with the group name, feces diameter, age, and sex group if known or deduced from fecal diameter, date, GPS location, appearance, and time. Livestock samples were gathered by routine visits into communities surrounding Bwindi Impenetrable National Park and taking fresh fecal samples from representative animals, while also assessing their age, health, sex, living conditions, history, date, time, appearance, and GPS location. Health assessments and management advice was also given at each village sampled, which was written up in SOAP format in the “Health Assessment” documents.

Fresh unpreserved gorilla and livestock samples were divided into formalin and frozen samples and formalin samples tested using the ImmunoSTAT method at the CTPH Gorilla Research Clinic in Buhoma.

### ImmunoSTAT Test

Field screening was implemented using the ImmunoSTAT Commercial Kit, which is a fecal antigen enzyme-linked immunosorbent assay test and Lateral flow immunochromatographic test with an embedded membrane to which antibodies raised against *Giardia* and *Cryptosporidium* sps. are immobilized to detect antigen A and B (25, 26). These were compared with positive controls, which came directly from the Manufacturer. This rapid assay test was used as a screening test for only *Giardia intestinalis* (*G. duodenalis, G. lamblia*) and *C. parvum* in fecal specimens. A treatment buffer was provided in the test kit, which was added to a small amount (1 g) of the fecal sample. An anti-Giardia biotinylated antibody reagent along with a monoclonal antibody colloidal dye suspension for both *Giardia* and *Cryptosporidium* were added.

In the field, samples were tested using the ImmunoSTAT Cards following the manufacturer's instructions. To prepare the samples, 1 g of feces was suspended in 3 ml of deionized water. The feces was macerated using a wooden applicator stick, the sample was thoroughly mixed, then 60 μl of the suspension was added to 2 drops of Sample Buffer, 2 drops of Reagent A, and 2 drops of Reagent B per the manufacturer's instructions. The mixture was then added to the ImmunoCard STAT sample well and then allowed to move via capillary motion into the testing area. The results were read 10 min later and compared to positive and negative controls. A black or gray line had to appear in the CONT position in order for the test to be valid and to ensure enough capillary motion had taken place. A “positive” was recorded when a black or gray line appeared at the CRYP or GIAR indicator and a “negative” was recorded if no line appeared at all. Although according to the manufacturer, a yellow or brown line at either the GIAR or CRYP position is considered invalid, it was decided to define these samples as “Negative Yellow” and re-test these samples using different techniques because of the large debris content in the animal samples.

Doubtful samples were tested with Direct Fluorescence Antibody Test of Fecal Samples for *Cryptosporidium* and *Giardia* that utilizes the principle of immunofluorescence to detect oocysts (27).

Frozen samples were carried to the Microbiology Laboratory, Faculty of Veterinary Medicine, Makerere University for DFA testing. Specimens kept at −20°C were sent to the Faculty to confirm the presence of *Cryptosporidium*/*Giardia* after the primary test became doubtful using immunocard STAT test at the Gorilla Research Clinic.

The Direct Fluorescent Antibody (DFA) test was performed after the specimens were stained using Modified Ziehl Neelsen (MZN) stain to test for *Cryptosporidium*. The DFA test uses fluorescently labeled monoclonal antibodies to bind and illuminate a target antigen. Samples were prepared by fixing on the slides. Fluorescent antigen was added and time was allowed for binding to occur. The unbound antigen was rinsed and examined under the microscope. If samples contained the antigen, they emitted light.

## Results

### Gorillas

Two habituated gorilla fecal samples tested using the ImmunoSTAT were positive for *Cryptosporidium* sp., one from a Habinyanja (H) gorilla group infant and one from a Rushegura (R) gorilla group infant, which range in Bujengwe and Mukono parishes, respectively. An additional doubtful sample tested positive for *Cryptosporidium* using the DFA test bringing the total positive samples to three (3.1%) habituated gorillas. No habituated gorillas tested positive for *Giardia* sp. None of the samples from the gorilla census tested positive for either pathogen.

### Livestock

Five livestock samples tested positive using the ImmunoSTAT for *Cryptosporidium* sp. while 4 samples tested positive for *Giardia sp*. Using the DFA test, one additional doubtful sample tested positive for *Cryptosporidium* and three for *Giardia* bringing the total positive cattle samples to 6 (4.7%) for *Cryptosporidium* and 7 (5.5%) for *Giardia*.

### Humans

**Figure 2:**
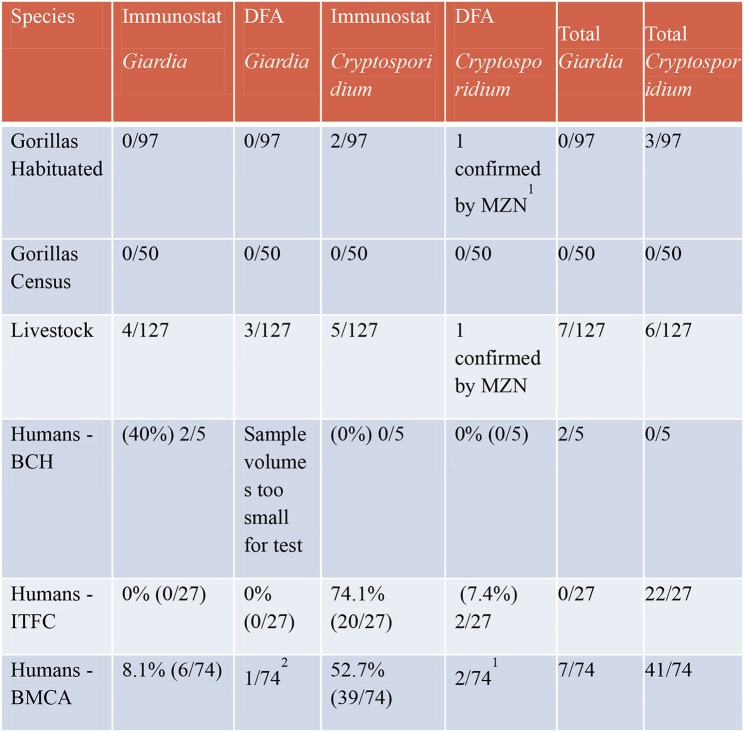
Results of ImmunoSTAT and direct immunoflorescent antibody tests for *Giardia* and *Cryptosporidium*.

Eight humans tested positive using the ImmunoSTAT for *Giardia sp*. and an additional doubtful sample tested positive for *Giardia* using the DFA test bringing the total positive human samples to 9 (8.5%). Fifty-nine humans tested positive using ImmunoSTAT for *Cryptosporidium* sp. and additional 4 doubtful samples were confirmed with the DFA test bringing the total to 63 (59.4%) positive human samples to *Cryptosporidium*. Figure 2 shows a summary table of these results.

## Discussion

### Prevalence

*Giardia* sp. was not found in either habituated or unhabituated gorillas, unlike previous studies (18, 22). Improvement in human health care and education where gorillas range into community land could be a contributing factor through a One Health program initiated by CTPH and efforts of other local community health service providers. Previous studies suggested that the *Giardia* Assemblage A genotype may have been introduced into gorilla populations through co-habituation activities with humans and other mammals and may have then been sustained in their habitats by anthropozoonotic transmission because a large percentage of the local community does not follow park regulations regarding the disposal of their fecal waste, as self-reported in a questionnaire (17, 22), which implied that the genotypes of *Giardia* samples isolated from gorillas in Volcanoes National Park in Rwanda, have been reported in humans, suggesting that the importance of humans in this ecosystem should be more closely evaluated.

*Cryptosporidium* sp. was found in 3.1% of gorillas and only in habituated gorillas. This is in agreement with the first study to report *Cryptosporidium* sp. infections in gorillas, which found that gorilla groups at Bwindi with closest proximity to people have the highest prevalence and may be a result of the habituation process and ecotourism (17).

The prevalence of *Giardia sp*. in symptomatic humans (five infants that had diarrhea at BCH) is relatively high at 40%, whereas non-symptomatic humans (park staff) had a prevalence of 7%. The prevalence of *Cryptosporidium sp*. in non-symptomatic humans was relatively high in both sets of staff who work with gorillas. Giardiasis typically results in severe diarrhea, but cryptosporidiosis is only associated with severe clinical signs typically in immunocompromised and malnourished patients. The high prevalence of *Cryptosporidium* sp. in park staff ranging from 55 to 82% indicates the need for a preventative health program for staff working with gorillas.

The prevalence of both pathogens from sympatric goats and cattle was relatively low ranging from 4.7 to 5.5%. Both goats and cows are grazed at the edge of the park and could be a source of infection for gorillas when they forage in community land. This has been implied in previous studies at Bwindi (17, 18).

### Comparison and Feasibility of the Tests

Similar to previous studies in humans, the DFA test in gorillas and livestock was more sensitive than the ImmunoSTAT test (25). Rapid Assays only detect the antigen, which may be shed as asexual stages of the organism while DFA detect intact oocysts shed in feces by the FIFC conjugate monoclonal antibody binding to surface exposed epitopes on the *Cryptosporidium* sp. oocysts. However, both methods are more reliable than direct microscopy for diagnostic work (27).

This study showed that a combination of both tests in gorillas and livestock had the same effects as human medicine making the protocol feasible for detection of *Cryptosporidium* and *Giardia* during wildlife and livestock disease investigations to increase laboratory efficiency by reducing labor, time and costs by screening samples under field conditions using the ImmunoSTAT, a rapid immunoassay and then confirming doubtful samples with a conventional Direct Fluorescent-antibody (DFA), which is a more sensitive test, but more sophisticated and labor intensive (25).

### Management Implications

The presence of *C. parvum* in gorillas is an indication of exposure to humans and livestock, as has been found in previous studies (17, 23). The two villages, Mukongoro and Kyogo, where two symptomatic human samples were positive for *Giardia sp*. and two asymptomatic gorilla samples were positive for *Cryptosporidium sp*., had the worst hygiene and sanitation indicators based on a household baseline survey conducted in September 2009. These villages are also in an area where gorillas from Rushegura gorilla group often pass while on the way to and from the Bwindi-Sarambwe border with Democratic Republic of Congo (DRC).

The poor health status of these communities prompted Conservation Through Public Health (CTPH) to select a second community conservation health volunteer (CCHV) or VHCT in Mukongoro and Kyogo villages in December 2009 to intensify hygiene and sanitation behavior change communication. CTPH also shared the results with BCH and recommended education and public outreach with patients regarding water collection from protected water sources. A baseline survey conducted in 2009 showed that only 53% of households collected water from protected water sources (CTPH, unpublished information). CTPH also taught farmers to build water troughs for their cattle to prevent them defecating in water sources shared by gorillas, people and livestock.

Molecular typing of both parasites in humans, domestic animals, and wildlife to date indicates a complex picture of both anthroponotic, zoonotic and spill-back transmission cycles that requires further investigation and a One Health approach to reduce cross species disease transmission (16).

## Conclusion

“One Health” is a terminology used to describe an approach that addresses human, animal and ecosystem health together. This paper has described elements of a One Health field program established by CTPH at Bwindi Impenetrable National Park to promote gorilla conservation through comparative pathogen analysis at the human/gorilla/livestock interface and implementing targeted management interventions in the human health and livestock health sectors. Conducting comparative pathogen analysis between people, wildlife and livestock presents the opportunity to provide timely information for more rapid management of disease by wildlife, veterinary, and public health managers.

## Author Contributions

GK-Z initiated the research topic, provided some funding, and supervised the research as a principal investigator. This involved contributing to design of the study, sample collection and analysis, interpretation of results, and implementation of management actions based on research findings, as well as drafting the manuscript. SR contributed to the design of the research, participated in sample collection and laboratory analysis, as well as results interpretation and implementation of management actions based on research findings. BM contributed to the design of the study and participated in sample analysis of people, interpretation of the results and writing of the manuscript. RS got the donation of fecal antigen test kits from the manufacturer, provided some funding, contributed to the design of the research, and conducted the research by participating in sample collection and analysis, and results interpretation.

### Conflict of Interest Statement

The authors declare that the research was conducted in the absence of any commercial or financial relationships that could be construed as a potential conflict of interest.
